# Sociodemographic and clinical characteristics of patients on Methadone followed at Ar Razi hospital in Morocco

**DOI:** 10.1192/j.eurpsy.2024.849

**Published:** 2024-08-27

**Authors:** Y. Bensalah, M. Sabir, F. Elomari

**Affiliations:** Psychiatric hospital Ar RAzi, Salé, Morocco

## Abstract

**Introduction:**

Opioid substitution treatments, notably methadone, now represent the standard treatment in the management of opioid dependence, making it possible to reduce illicit opioid consumption, crime, infections linked to administration practices and improve socio-professional integration

**Objectives:**

Determine the socio-demographic and clinical characteristics of the patients Evaluate the quality of life of these patients

**Methods:**

A cross-sectional, descriptive and analytical study was conducted with 60 patients receiving methadone followed at Ar-Razi Hospital in Salé between 01 june 2023 and 30 august 2023. A questionnaire was used to assess the socio-demographic and clinical characteristics of the patients. Quality of life was assessed using the 36-item Short Form Health Survey SF-36 scale

**Results:**

The average age of our patients was 34 years with a male predominance Most of our patients were single and unemployed Somatic disorders were found in 15% of the sample The majority of them had an associated depressive disorder The main types of new psychoactive substances consumed were benzodiazepines (62.3%) and cannabis.

Quality of life was impaired in 60% of patients treated with methadone

**Conclusions:**

The population using methadone is precarious and presents somatic and psychiatric vulnerability. Forms of misuse and associated consumption of other psychoactive substances and illicit drugs are recorded, hence the need for early detection in order to improve care

**Disclosure of Interest:**

None Declared

